# Integrated Diagnosis in Africa’s Low- and Middle-Income Countries: What Is It, What Works, and for Whom? A Realist Synthesis

**DOI:** 10.5334/ijic.7788

**Published:** 2024-09-12

**Authors:** Gamuchirai Gwaza, Annette Plüddemann, Marcy McCall, Carl Heneghan

**Affiliations:** 1 Department for Continuing Education, University of Oxford, United Kingdom; 2 Nuffield Department of Primary Care Health Sciences, University of Oxford, United Kingdom

**Keywords:** integrated diagnosis, primary healthcare, Africa, realist synthesis

## Abstract

**Introduction:**

Integrated diagnosis can improve health outcomes and patient experiences through early diagnosis and identification of cases that could otherwise be overlooked. Although existing research highlight the feasibility of integrated diagnosis across various conditions, a significant evidence gap remains regarding its direct impact on patient experiences and health outcomes. This review explores the conceptualizations of integrated diagnosis by different stakeholders along the healthcare pathway and examines the necessary contexts and mechanisms crucial for its effectiveness.

**Methods:**

This study adopts a realist methodology to explore integrated diagnosis. Using a systematic approach, the research aims to collect, assess, and synthesize existing evidence on integrated diagnosis, guided by a program theory developed through literature review and expert consultations. Primary studies and reviews related to integrated diagnosis, multi-disease testing, or integrated healthcare with a diagnostic focus were sourced from major databases and global health organization websites. The collected evidence was used to construct and refine the evolving theoretical framework.

**Results:**

This study identified three models of integrated diagnosis interventions: individual/human resource integration, facility or mobile-based integration, and technology integration. Successful implementation of these models relies on understanding the values and perceptions of both healthcare workers and patients/clients. This research emphasizes a holistic approach that considers all elements within the health system and underscores their interdependence. Using the WHO health systems framework to contextualise factors, the study positions diagnosis as an integral component of the broader health ecosystem. A key finding of the research is the importance of addressing the barriers and facilitators of integrated diagnosis interventions. This includes policy frameworks, diagnostic tools, funding mechanisms, treatment pathways, and human resource issues. Improving patient experiences requires cultivating positive relationships with healthcare workers ensuring elements such as respect, confidentiality, accessibility, and timeliness of services are prioritised.

**Discussion and Conclusion:**

The diverse conceptualisations of integrated diagnosis highlight the importance of clear definitions for each intervention. This clarity is essential for transferring lessons learned, comparing programs, and effectively measuring results. The success of integrated diagnosis is not a one-size-fits-all scenario; decisions regarding the approach, conditions to be integrated, and timing of integration must be guided by local contexts to ensure sustainable outcomes. The review findings suggest that integrated diagnosis may be suitable at the primary care level in LMICs under specific circumstances. Successful implementation hinges on addressing the perspectives of healthcare workers and patients/clients alike, requiring adequate time, resources, and a well-defined intervention model.

## Introduction

This review explores the concept of integrated diagnosis, a component of integrated health care, examining its effectiveness across various conditions and populations. While there are some widely accepted definitions of integrated health care, its interpretation often varies based on specific contexts, as no single definition universally fits all scenarios [1,2]. According to the World Health Organisation (WHO), integrated health services are services that are managed and delivered in a way that ensures people receive a continuum of health promotion, disease prevention, diagnosis, treatment, disease management, rehabilitation, and palliative care services at the different levels and sites of care within the health system, and according to their needs throughout their life course [3]. Diagnosis constitutes a crucial step in this care cascade.

The diagnostic process involves a range of activities, including clinical history and interview, physical examination, testing, and consulting with other clinicians [4]. Integrated diagnosis, in turn, can encompass various forms: from diagnostics that address multiple diseases or conditions (such as multi-disease or multi-analyte platforms) to interventions targeting individuals with co-infections such as TB and HIV or leveraging existing health programs for new diagnostic purposes [5,6]. These variations in conceptualisation complicate the comparison, replication, evaluation, and scalability of integration models across different settings, contributing to knowledge gaps in understanding their true impact [7,8,9]. Moreover, the review explores the contextual factors and mechanisms necessary for integrated diagnosis to improve patient experiences and health outcomes effectively.

### Rationale for the Review

Africa still has the highest disease burden for many communicable and non-communicable diseases (NCDs) [10]. Failure to control communicable diseases is, in part, due to challenges in correctly diagnosing and treating cases, especially at primary care facilities. For instance, a significant number of new tuberculosis (TB) cases and people living with HIV remain undiagnosed, missing opportunities for early intervention and prevention [4,11]. Many adults are unaware of their NCD status [12]. As life expectancy improves for individuals with HIV due to treatment, there is an increasing risk of comorbidity with NCDs [13]. It is estimated that reducing the diagnostic gap for the six tracer conditions for general health system performance, that is, HIV, TB, diabetes, hypertension, syphilis, and hepatitis B virus infection, could significantly reduce premature deaths and disability-adjusted life-years in LMICs [4]. As such, there is a need for improved healthcare delivery models to ensure that patients are not missed or lost to follow-up.

There is an increasing level of political recognition of the importance of an integrated approach to healthcare delivery [14]. The Organization for Economic Cooperation and Development (OECD) sees integration as a quality indicator [8]. In 2016, WHO developed integrated healthcare guidelines, which encompass various healthcare processes, including integrated diagnosis [15] though the utilisation of the Integrated people-centred health services framework remains limited [16]. In 2019, WHO recommended the development of integrated testing services and policies, and there have been policy guidelines for integrating conditions such as TB, HIV, and NCDs, where integrated diagnosis can be beneficial [17,18,19]. In May 2023, the World Health Assembly adopted a resolution to ‘Strengthen Diagnostics Capacity,’ highlighting the vital role of diagnostics in health programming and pointing to key gaps needing coordinated action from global and national partners [20]. The resolution recommends breaking down silos through more integrated approaches to screening and diagnosis and decentralizing testing to point-of-care in both primary care settings and the community level. However, a growing body of literature indicates that the policy focus on integration may not align with the complex realities of service delivery in LMICs [21].

### Objectives and Focus of the Review

The primary objective of the review is to explore what integrated diagnosis means for different stakeholders and the specific contexts and mechanisms necessary for the successful implementation of integrated diagnosis interventions. Success in this context is defined by the achievement of intended outcomes, that is, improved patient experiences and health outcomes at the primary care level in LMICs, particularly in Africa.

## Methods

The realist approach is used, which offers tools for synthesising complex evidence from interventions [22,23,24]. The realist review uses systematic, theory-driven interpretative techniques to help make sense of heterogeneous evidence about complex interventions applied in diverse contexts in a way that informs policy [23,24]. Realist philosophy borrows and builds on both the positivist and constructivist paradigms. On the one hand, like positivism, realism believes we can objectively measure and observe the real world. However, like constructivism, we interpret our reality through a subjective lens, such as culture, and therefore, we can only measure it indirectly [25]. As such, though the individual agency is involved, realism posits that individuals are likely to make similar choices about resource utilisation in specific contexts, leading to semi-predictable patterns of behaviour. The realist seeks to uncover these patterns by examining the interaction between context, mechanisms, and outcomes (C-M-O). Mechanisms are the underlying entities, processes, or social structures that operate in specific contexts to generate the desired results [24].

A rapid literature review was conducted to develop a theoretical framework for analyzing integrated diagnosis interventions, which guided expert consultations. The literature search was comprehensive and iterative, covering academic databases and global health organizations’ reports. Studies were included if they addressed multi-disease diagnosis at the primary care level in LMICs. Data was extracted, coded, and analyzed using NVivo software, focusing on context, mechanism, and outcome (C-M-O) elements. The analysis aimed to test and refine theories, identifying patterns that either supported or challenged the initial program theories, ensuring a robust understanding of integrated diagnosis interventions.

## Results

### Document Characteristics

A total of 25 primary studies published between 1984 and 2022 were analyzed, encompassing a variety of research designs. Overall, the studies were implementation research [34,35,36,37,38,39,40], qualitative studies [13,41,42,43,44], program evaluations [45,46,47], observational studies [48,49] and controlled trials [50,51]. Of these, 76% involved HIV as one of the integrated diseases. Additionally, 15 systematic reviews were included covering topics such as general health service integration [9,26]; patient experiences of integrated care [27,28]; TB and HIV integration [29]; Integrated Management of Childhood Illnesses (IMCI) [30]; HIV and NCDs [7,14,31,32,33,34], and sexual and reproductive health [35]; antenatal health services [36]; and NCDs and reproductive, maternal, newborn and child health [37]. Supplementary file 1 lists the primary studies and systematic reviews that have been included.

### Main findings

**Figure 1:**
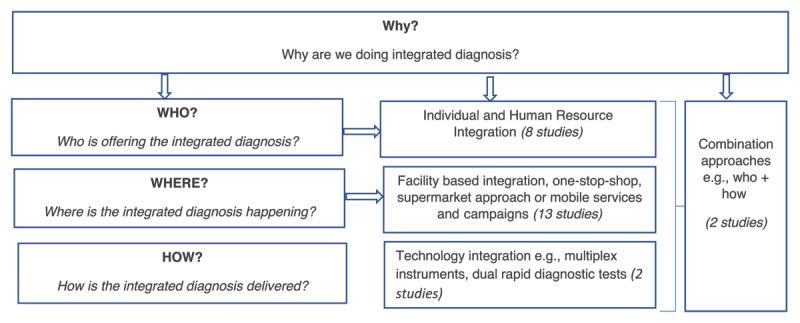
Ways that integrated diagnosis was conceptualised.

The first question in the review examined how different stakeholders conceptualised integrated diagnosis, revealing that interpretations varied based on the intervention’s focus, the service provider, the implementation setting, and the method of integration (Figure 1). Integrated diagnosis was frequently viewed narrowly, focusing primarily on testing procedures rather than the broader diagnostic process. For example, many primary studies centered on near point-of-care or point-of-care tests for diseases like HIV, TB, or syphilis, using technologies such as dual Rapid Diagnostic Tests (RDTs) or GeneXpert. This approach often limited integrated diagnosis to multi-disease testing, with minimal patient examination beyond sample collection unless test results were positive.

#### Types of integrated services

The WHO defines integrated healthcare as a coordinated approach that ensures individuals have access to multiple healthcare services based on their specific needs. The ultimate aim is to enhance patient experiences and improve health outcomes [3,38]. While the WHO outlines the purpose and objectives of integrated healthcare, it does not prescribe the specific actors, locations, or methods for integration, providing general guidance to facilitate the implementation of integrated healthcare.

The review identified three types of integrated diagnosis interventions:

**I. Facility Integration:** This approach involved providing diagnosis for different conditions in a single physical location, often referred to as the “supermarket approach” or “one-stop shop” [7,12,39,40,41,42,43,44,45]. It could include co-located services within the same facility or referral-based services to different buildings. Examples include integrated TB and HIV integration [42], NCD and HIV testing in South Africa [12], and a Chronic Care Clinic model in Malawi [46].
  Facility integration, often used to combine traditionally “vertical programs” into integrated services, is endorsed by global health organizations like UNAIDS to enhance health outcomes by leveraging HIV programs. [5,47]. Facilities with regular healthcare touchpoints, such as maternal health services for pregnant women, offer convenient opportunities to integrate other essential diagnostic services including for sexually transmitted infections (STIs), cervical cancer, and hypertension. [37,39,40,44,45]. Additionally, targeting key populations can improve testing coverage, particularly in cases with high co-infections rates, such as HIV and TB [48].
  **Mobile and Campaign-Based Integration**, a subtype of facility-based integration involves using mobile clinics or health campaigns to offer integrated diagnostic services [32,49,50,51], such as HIV and NCD testing in countries like South Africa and Malawi [46,49]. A significant challenge with this approach is the poor linkage to care, where many individuals who receive diagnoses during these initiatives do not follow through with necessary treatment and care. For instance, in a mobile screening effort in Lesotho, only 36.6% of those who tested positive for HIV enrolled in further care [7]. However, patient-centered strategies, like a hotline for support and reminders for clinic visits have been shown to improve these linkages [52].
**II. Individual or Human Resource integration** involves patients consulting with a single healthcare provider who delivers multiple diagnostic services during one visit typically within a single facility [30,41,53,54,55,56,57,58]. Unlike facility integration, which involves multiple professionals, individual integration relies on one provider to manage various services. This model is often used for case management in programs like IMCI or for conducting symptomatic diagnoses [30,59].
**III. Technology Integration** in healthcare focuses on using technology to deliver integrated diagnostic services, such as multi-disease testing platforms for TB and HIV testing [60,61]. The aim is to improve test result turnaround time, optimise technology use, and reduce program costs [62]. However, a significant risk with this approach is that it may become instrument-centered rather than patient-centered. For example, while technologies like GeneXpert emphasize accuracy and speed, they can lead to more transactional consultations, potentially neglecting patient comfort, comprehensive counseling, and overall healthcare experience.
  **Combination Approaches** in healthcare integration blend different types of integration models. For example, a facility based approach for HIV and TB might involve a single healthcare provider diagnosing both diseases [63]. This model streamlines the process, enabling quicker initiation of treatment by combining the benefits of both facility and individual integration.

**The patient definition of integrated diagnosis** includes two main concepts:

**Single Provider:** Patients view integrated diagnosis as receiving comprehensive, continuous care for multiple conditions from a single healthcare provider. They prefer holistic care that addresses all their health needs together, rather than treating each condition separately. For example, in South Africa, patients with comorbidities saw their conditions as interconnected and preferred receiving care from one professional who could address their overall chronic suffering [13]. Despite this preference, global health agencies like the Global Fund and PEPFAR often focus on specific diseases like HIV and TB, though there is a move towards integrating these vertical approaches to offer more cohesive care.

**Convenient Access to Multiple Services:** Patients also define integrated care as having easy access to multiple healthcare services within the same facility. Even when services for different conditions are offered on different days, patients may still perceive them as integrated because they are accessible in one location [64]. Patients considered these services integrated even though the programs were not structurally or functionally integrated.

#### Contexts and Mechanisms necessary for effective interventions

The second question in the review aimed to identify the contexts and mechanisms essential for the success of integrated diagnosis interventions. Given the complexity of integrated diagnosis, a health systems approach is necessary to understand the factors contributing to its success. Healthcare workers (HCWs) and patients have distinct perspectives and motivations in the healthcare process, necessitating the development of separate theories or C-M-O (Context-Mechanism-Outcome) models to better understand their unique roles and participation in the diagnostic process.

##### Health Care Workers (HCWs)

**Figure 2:**
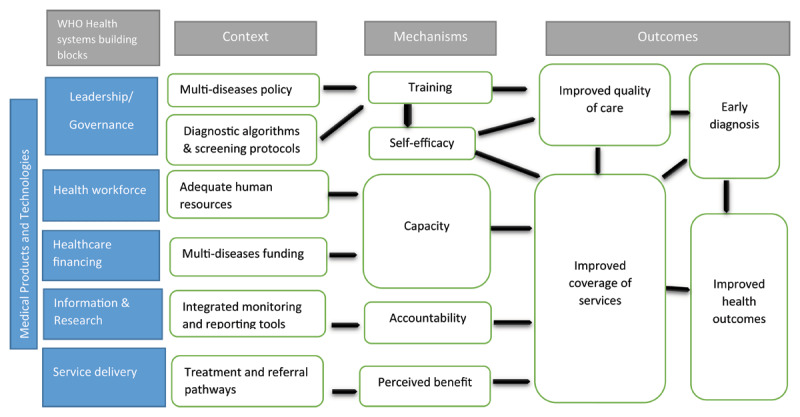
C-M-O configurations for healthcare workers with a mapping of how they align with the WHO health systems framework.

The WHO Health Systems building blocks have been used as an analytical framework to identify the various contexts required for successful implementation [65]. These building blocks include service delivery, health workforce, health information systems, access to essential medicines, health financing, and leadership and governance. Prioritising these elements allows healthcare systems to strive for equitable, affordable, and high-quality care for all. Figure 2 summarises the contextual factors from the studies that align with the six WHO building blocks for health systems.

**a. Leadership and Governance**

This building block focuses on the establishment of strategic policy frameworks, effective oversight, accountability, regulations, and incentives [65].

**Multi-disease policy:** The presence of global or national multi-disease policies is a key to designing successful integrated healthcare interventions [42]. Many LMICs rely on WHO guidelines to shape their national policies and donor priorities. For example, the WHO guidelines on NCD integration have led to increased national prioritisation and funding for NCD interventions [33]. In some cases, integrated diagnosis interventions began before formal policies were established, as seen with cervical cancer programs, where funding and training motivated healthcare workers despite the absence of a national cancer integration strategy [39].

**Diagnostic Algorithms and screening protocols:** The effective implementation of multi-disease policies relies on well-developed diagnostic algorithms and screening protocols. For example, the successful integration of HIV and TB in many LMICs is due to clear WHO guidelines, national policies, operational plans, healthcare worker training, and adequate funding [29]. However, when such policies are not properly operationalized, the implementation can be inconsistent. In Botswana, for example, the lack of national clinical guidelines for NCDs before 2016 led to inconsistent care and referrals for conditions like diabetes and hypertension, depending on the healthcare worker’s training [66]. Even when national policies support service integration, such as HIV and SRH, gaps and inconsistencies often arise due to a lack of standardization [35]. Without simple screening or diagnostic algorithms, healthcare workers may hesitate to act, fearing diagnostic errors or potential harm to patients [36].

**Training:** Training is essential for effective use of policies, guidelines, and resources, as it significantly influences the self-efficacy, engagement and job satisfaction of healthcare workers [67]. Proper training enables HCWs to provide holistic care, as seen in Mozambique and Kenya, where it improved job satisfaction and effectiveness in integrated healthcare settings [36]. In Tanzania, training in IMCI led to better quality of care, with trained HCWs delivering more accurate assessments and treatment of children [30]. Training should encompass technical skills, interpersonal relationships, and patient-centered care, as interprofessional collaboration is vital for successful facility integration [68]. While training can be resource-intensive, requiring ongoing support and potentially leading to reduced service quality if HCWs are taken away from frontline duties. Additionally, if disease prevalence is low, HCWs may stop testing, leading to a loss of competencies that require regular practice [19,36].

**b. Health Workforce**

The availability of a sufficient and responsive health workforce is critical to the quality and delivery of healthcare services [65]. Ensuring that the workforce has the necessary material and human resource is essential to prevent integration from negatively impacting staff or patients [5]. The success of integrated services largely depends on HCW skills and their adherence to the intervention protocols.

**Capacity for integrated diagnosis** is essential, requiring the health workforce to develop the skills and knowledge needed to manage multiple targeted conditions. Staff at all levels must acquire new expertise in the additional diseases they are expected to manage [69]. A review of integration efforts in maternal healthcare, combining services like HIV, tuberculosis, malaria, syphilis, and nutrition services with antenatal care, revealed concerns about care pathways [70,71]. HCWs sometimes avoided interventions out of fear of causing harm, rather than risking potential errors.

**Adequate human resources:** HCWs expressed concerns about increased workload and pressure when integrating additional tasks like routine HIV testing or TB screening into their responsibilities, especially with no additional compensation [36,72]. To manage staff workload, the Integra initiative in Kenya and Swaziland implemented strategic human resources planning, allocating specific days for different patient groups. For example, pregnant women were seen on Wednesdays, while patients with HIV and comorbid conditions attended a weekly ‘dual diagnosis day.’ [32]. However, it’s worth noting that integrated sites didn’t consistently yield better health outcomes; results were often mixed [21]. At the Epako clinic in Namibia, setting specific days for antiretroviral (ARV) clinics led to stigma, as it identified HIV-positive individuals. Integration helped by offering all services daily, which reduced stigma [73].

**c. Healthcare financing**

Adequate health financing is essential for the success of integrated diagnosis interventions, ensuring services are accessible while protecting individuals from financial hardship [65]. Multi-disease funding is crucial but must be well-coordinated to avoid negative consequences. For example, in Lesotho, poor coordination among donors funding TB and HIV programs with different reporting requirements hindered integration efforts [74].

In many LMICs, donor funding often targets specific diseases like HIV, leading to neglect of other conditions. This imbalance can create disincentives for HCWs, leading to inefficiencies and dissatisfaction when certain diseases receive more funding and incentives [32,35].

Unbalanced funding can also impact the sustainability of health programs, as HCWs may lose motivation if incentives are reduced or withdrawn. In some cases, patients receive incentives to encourage participation in services like HIV and NCD screening, promoting early diagnosis and care-seeking behavior [49]. However, charging user fees to address funding gaps can limit service uptake, especially among financially disadvantaged populations [21].

**d. Information and research**

The health information system is vital for producing, analysing and using reliable health data, which is crucial for informed decision making in health interventions [65] [71].

**Integrated Monitoring and Reporting Tools** such as standard templates and registers covering all targeted diseases, are essential for the success of integrated diagnosis interventions. These tools help HCWs remain accountable and promote a holistic approach to healthcare. However, when integrated indicators are lacking, as seen in the integration of HIV and SRH services, it can lead to separate and inefficient reporting systems [5,58]. It’s important that these tools remain simple to encourage HCWs to use them effectively [32]. Digital innovations can further streamline reporting processes, reducing paperwork, and allowing HCWs to focus more on patient care. Additionally, provider job aids like screening tools and flipcharts, have proven effective in supporting integrated diagnosis activities [41].

**e. Medical Products/Technologies**

The availability of health technologies, equipment, and a reliable supply chain is critical for delivering quality healthcare services [65]. A major challenge to the success of integrated diagnostic services, such as those for HIV, syphilis, and malaria, is the unavailability of essential commodities and irregular supplies [36,71]. Efficient supply chain management is essential to prevent stockouts and ensure that supplies are delivered on time, which requires robust logistics systems and partnerships with dependable suppliers. Adequate financial resources must be allocated not only for the initial purchase of equipment but also for ongoing costs like consumables and maintenance to sustain integrated diagnostics services effectively.

**f. Service delivery**

Service delivery is a crucial element of the healthcare system, involving the provision of effective, safe, and high-quality health interventions supported by the necessary infrastructure. It ensures that health services are accessible to those in need at the right time and place while making efficient use of available resources [65]. The success of service delivery relies on the effective collaboration of all other healthcare system components. Furthermore, the experience and perception of patients in receiving these services are vital to the overall effectiveness and quality of care.

**Referral and Linkage to care**

Successful integrated diagnosis interventions depend not only on accurate diagnosis but also on effective referral and linkage to care. Well-defined treatment pathways, disease management strategies, and referral mechanisms are crucial, enhancing HCW engagement and building patient trust. For instance, in Kenya, the availability of treatment for conditions like HIV and other STIs increased testing uptake [41]. However, challenges arise when treatment capacity is inadequate, as seen in some NCDs integrations where ongoing diagnosis was unsustainable due to lack of treatment capacity [7,43]. Referral mechanisms can still pose issues even with functioning diagnosis and treatment services. A pilot in Malawi integrating hypertension screening into HIV services highlighted the need for better linkage to care for hypertension management [75,76].

In contrast, a cluster randomized trial in Uganda and Kenya demonstrated successful strategies for improving linkage to care, involving multi-disease care delivery and patient-centered treatment. This trial showed that interventions like mobile health campaigns, personalized care, flexible clinic hours, and proactive communication significantly improved clinical outcomes and reduced mortality rates related to HIV, TB, and hypertension [52].

##### Clients and Patients accessing diagnostic services

Community awareness, demand, and acceptability are crucial for the success of integrated diagnosis interventions. Patient-centered approaches, like the “Caring About Me” framework [77], emphasise the importance of healthcare providers collaborating with patients to address individual needs and support self-management.

**Figure 3:**
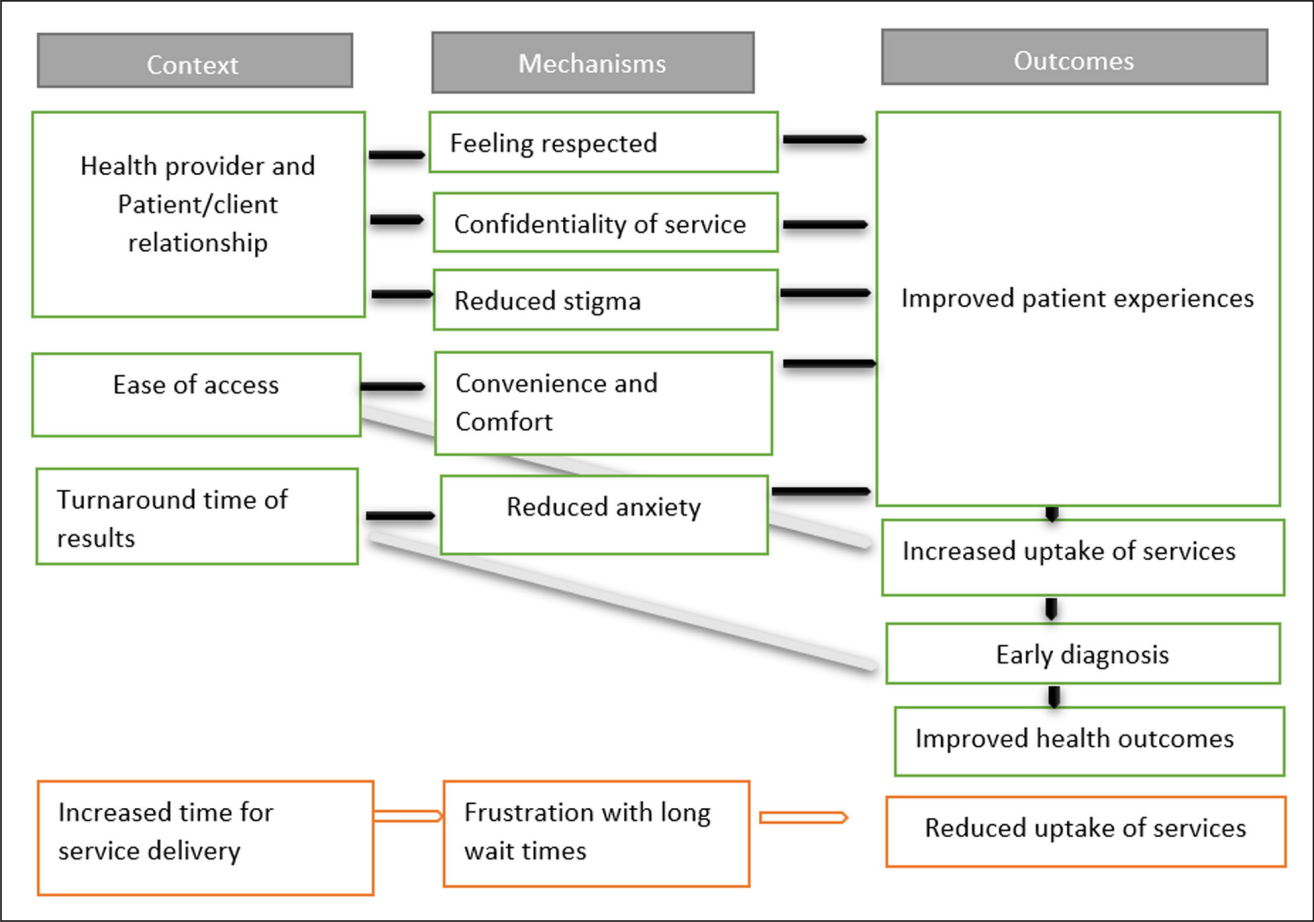
C-M-O configurations for patients to ensure successful integrated diagnosis interventions.

In LMICs, the focus of integrated diagnosis may shift from having individualized care to fostering a supportive, respectful, and non-judgmental environment. For example, SRH interventions that create such environments tend to lead to positive patient experiences, enhancing the overall success of integrated diagnostic services (Figure 3).

**a) Health provider and Patient/client relationship**

Patients who feel respected by healthcare workers (HCWs) tend to report more positive experiences, highlighting the importance of training HCWs in patient-centered care [41] [32]. Confidentiality is key to reducing the stigma associated with diseases like HIV and cancer. For example, in Namibia, services were provided in a way that maintained patient privacy, preventing others from knowing who was HIV positive [21].

However, privacy concerns can still arise, as seen in Swaziland, where inadequate privacy in healthcare facilities led to patient stress and reluctance to discuss personal matters during HIV testing. [21]. While provider-initiated diagnosis has increased testing uptake, it can also create power dynamics that make patients feel pressured to accept diagnoses, fearing negative consequences if they refuse. For instance, in some cases, nearly 29% of patients felt unable to decline HIV testing offered by TB nurses due to concerns about future care [72].

**b) Ease of access to services**

Ease of access to healthcare services is significantly influenced by the physical layout of the facility. Key aspects include having private consultation rooms, comfortable waiting areas, and clear access to the services [21]. Sometimes, separating different areas within the facility may be important to prevent disease transmission. For instance, in Kenya, combining HIV testing with SRH services led to lower testing rates due to concerns about HIV exposure [41].

**c) Turnaround time of results**

Faster delivery of test results greatly enhances patient experience by reducing waiting times, which can be stressful [78]. Same-day results boost patient confidence in the healthcare system. For instance, the integrated point-of-care testing reduced turnaround times for HIV testing from days to hours, improving clinical decision-making, quality of care, and health outcomes [12,78,61].

**d) Increased time for service delivery**

Timely service delivery improves health outcomes and convenience, particularly for conditions needing prompt treatment. However, integrating services can sometimes lead to longer wait times, causing frustration and reducing service uptake. For example, in a Zambian clinic, integrating HIV services with primary healthcare increased waiting times from 91 to 127 minutes [79]. Additionally, HCWs trained in IMCI took nearly two minutes longer per consultation, which sometimes made patients reluctant to discuss multiple health concerns, potentially impacting the quality of care [30] [21].

## Discussion

Integrated diagnosis is viewed differently by various stakeholders based on its purpose, participants, technology, and location. Patients prioritise convenience, confidentiality, reduced stigma, and respectful relationships with providers.

While integrated diagnosis can improve service uptake, especially among underserved groups, challenges arise in balancing quality service with limited staff and high patient volumes in LMICs. There’s a tension between improving patient experience, expanding access, and reducing costs, which requires strategic trade-offs. Integrated diagnostics can streamline healthcare by consolidating tests and visits, potentially saving costs through optimized resource use. However, initial setup and ongoing operational costs for equipment, consumables, and training can be significant.

The review revealed that challenges in integrated diagnosis interventions are complex and context-dependent, underscoring the need for tailored approaches. The Context-Mechanism-Outcome (CMO) heuristic can help in designing effective interventions by informing practice and decision making. Additionally, the realist review identified two key categories of explanatory theories relevant to HCWs and patients.

To effectively implement integrated diagnosis interventions, healthcare workers (HCWs) must adopt a health systems perspective, recognizing that changes in one part of the system can impact others. For instance, enhancing diagnostic capacity for specific diseases might strain resources and shift focus away from other services. The approach should consider all aspects of integration—structural, functional, social, and process—and ensure that all relevant disease programs, funding mechanisms, and diagnostic tools are coordinated. Effective implementation depends on adequately trained and motivated HCWs and comprehensive consideration of the entire health system, not just diagnostics. Additionally, patient confidence relies on the availability of resources, including treatments, which are often concentrated in secondary care settings rather than primary care in LMICs.

Integration should not replace addressing fundamental issues within struggling health systems [38,45]. Integrating services with varying efficiency levels is challenging and can lead to uneven outcomes. For instance, healthcare workers in one facility might struggle with additional tasks, while a similar facility in the same country might handle them more effectively. This variability greatly affects the success of integration interventions. [73,80]. [21].

Broader structural issues also impact integrated diagnosis interventions. Although donors and governments support integration, they often focus on disease-specific outcomes and partnerships, which can create contradictions and overlook systemic problems [81]. Implementation challenges include inadequate regulations, lack of multi-disease policies, and poor coordination among donors, leading to fragmented financing for integrated services. For instance, local regulations might not support integration, limiting the types of tests available at primary care levels.

Patient perspectives are crucial for improving healthcare experiences. Patients prioritize relationship-based care, confidentiality, ease of access, and timely services—factors not always addressed by structural integration efforts. Integration can sometimes lead to longer wait times, which may discourage patients from seeking care and affect their ability to express their needs.

Building a strong evidence base for integrated diagnosis is challenging because most interventions are small-scale, proof-of-concept studies that haven’t been scaled up. Integration models are new and lack comprehensive data on clinical outcomes, processes, and cost-effectiveness, limiting the assessment of their long-term effects. Additionally, many integration programs rely heavily on the HIV program as a model, which can overshadow and diminish the perceived importance of other healthcare programs.

In LMICs, facility integration is more feasible at the secondary care level, such as district hospitals because they are better resourced. Primary care facilities, such as clinics and health posts, face challenges like limited staff and space. These smaller facilities often struggle with maintaining privacy during consultations while managing high patient volumes. Individual integration in these settings is difficult due to high time and resource demands, which can cause patient frustration and service delays. Decision-makers must carefully weigh the benefits of individual integration against the challenges of providing timely and high-quality care.

When promoting integrated diagnosis approaches, it’s crucial to address the risk of over-diagnosis [82]. Offering additional diagnostic tests can lead to identifying more conditions than necessary, potentially resulting in unnecessary treatments or interventions. There is also a concern about intentional over-diagnosis driven by financial incentives, especially in settings with significant out-of-pocket costs. While integrated diagnosis improves accessibility and efficiency, it must be managed carefully to avoid excessive testing and maintain patient-centered care. Strategies to mitigate these risks include establishing clear test guidelines, ensuring transparency in diagnostic processes, and implementing safeguards against unnecessary tests. These measures are vital for optimizing healthcare delivery and ensuring equitable access to quality diagnostic services in LMICs.

## Limitations and future research direction

The review was conducted by a single reviewer, which might lead to the omission of important studies; however, expert feedback was used to address this issue.

Future research should focus more on integrated diagnosis and multi-disease testing, with longer-term studies involving larger populations to better assess their impact on patient healthcare experiences. Many reviews on integrated healthcare do not specifically address integrated diagnosis or multi-disease testing in detail. Existing studies often lack thorough explanations of the interventions and their mechanisms. Adopting a realist approach can help clarify how integrated diagnosis functions and identify key elements that enhance patient experiences and health outcomes.

## Conclusion and Practical Implications

The review focused on integrated diagnosis interventions primarily in Africa, with potential relevance to LMICs globally. Success in integrated diagnosis depends on tailoring the approach to local contexts, including specific conditions and timing, to ensure sustainable outcomes. Effective implementation requires addressing both healthcare workers’ and patients’ needs, which demands sufficient time, resources, and a clearly defined intervention model.

Recommendations include:

- Apply systems thinking when designing integrated diagnosis intervention, with a comprehensive view of the entire care cascade.
- Interventions should be informed by evidence. Analyze and interpret existing data to inform interventions, ensuring that reporting tools cover all conditions and integrate with existing systems.
- Emphasize clear and transparent documentation in research and programs to improve understanding of different integration models and facilitate meaningful comparisons and learning.

## Additional File

The additional file for this article can be found as follows:

10.5334/ijic.7788.s1 Supplementary file 1. List of primary studies included in the review.

## References

[1] Goodwin N. Understanding Integrated Care. Int J Integr Care. 2016; 16(4): 6. DOI: 10.5334/ijic.2530 PMC5354214 28316546

[2] Frisch NC. What’s in a Definition? Holistic Nursing, Integrative Health Care, and Integrative Nursing. Journal of Holistic Nursing. 2019; 37. DOI: 10.1177/0898010119860685 31257971

[3] WHO. WHO global strategy on people-centred and integrated health services; 2015. 10.1111/ajr.12209 26009975

[4] Fleming KA, Horton S, Wilson ML, Atun R, DeStigter K, Flanigan J, et al. The Lancet Commission on diagnostics: transforming access to diagnostics. Lancet. 2021; 398(10315): 1997–2050. DOI: 10.1016/S0140-6736(21)00673-5 34626542 PMC8494468

[5] Sweeney S, Obure C, Maier C, Greener R, Dehne K, Vassall A. Costs and efficiency of integrating HIV/AIDS services with other health services: a systematic review of evidence and experience. Global Health and Development; 2011. DOI: 10.1136/sextrans-2011-050199 22158934

[6] Unitaid. Multi-Disease diagnostic landscape for integrated management of HIV, HCV, TB and other coinfections; 2018.

[7] Haldane V, Legido-Quigley H, Chuah FLH, Sigfrid L, Murphy G, Ong SE, et al. Integrating cardiovascular diseases, hypertension, and diabetes with HIV services: a systematic review. AIDS Care. 2017; 30(1): 103–15. DOI: 10.1080/09540121.2017.1344350 28679283

[8] Trankle SA, Usherwood T, Abbott P, Roberts M, Crampton M, Girgis CM, et al. Integrating health care in Australia: a qualitative evaluation. BMC Health Serv Res. 2019; 19(1): 954. DOI: 10.1186/s12913-019-4780-z 31829215 PMC6907151

[9] Dudley L, Garner P. Strategies for integrating primary health services in low- and middle-income countries at the point of delivery. Cochrane Database Syst Rev. 2011(7): CD003318. DOI: 10.1002/14651858.CD003318.pub3 21735392 PMC6703668

[10] Sentes K, Kipp W. Global Burden of Disease: Huge Inequities in the Health Status in Developing and Developed Countries. Brief report; 2003. DOI: 10.1016/S0840-4704(10)60229-3 14618830

[11] Umubyeyi Nyaruhirira A, Scholten JN, Gidado M, Suarez PG. Coronavirus Disease 2019 Diagnosis in Low- and Middle-Income Countries: The Big New Bully Disrupting TB and HIV Diagnostic Services. J Mol Diagn. 2022; 24(4): 289–93. DOI: 10.1016/j.jmoldx.2021.12.008 35123038 PMC8810266

[12] Hopkins KL, Hlongwane KE, Otwombe K, Dietrich J, Cheyip M, Olivier J, et al. The substantial burden of non-communicable diseases and HIV-comorbidity amongst adults: Screening results from an integrated HIV testing services clinic for adults in Soweto, South Africa. EClinicalMedicine. 2021; 38: 101015. DOI: 10.1016/j.eclinm.2021.101015 34308316 PMC8283339

[13] George S, McGrath N, Oni T. The association between a detectable HIV viral load and non-communicable diseases comorbidity in HIV positive adults on antiretroviral therapy in Western Cape, South Africa. BMC Infect Dis. 2019; 19(1): 348. DOI: 10.1186/s12879-019-3956-9 31029087 PMC6487071

[14] Haregu TN, Setswe G, Elliott J, Oldenburg B. Integration of HIV/AIDS and noncommunicable diseases in developing countries: rationale, policies and models. International Journal of Healthcare. 2015; 1(1). DOI: 10.5430/ijh.v1n1p21

[15] Organisation WH. Framework on integrated, people-centred health services; 2016.

[16] Hafiz O, Yin X, Sun S, Yang J, Liu H. Examining the Use and Application of the WHO Integrated People-Centred Health Services Framework in Research Globally – a Systematic Scoping Review. Int J Integr Care. 2024; 24(2): 9. DOI: 10.5334/ijic.7754 PMC11049668 38681978

[17] WHO, ASLM. Molecular Diagnostics Integration Global Meeting Report, 10–12 July, Geneva, Switzerland; 2019. https://www.who.int/publications/i/item/9789240002135.

[18] WHO. WHO policy on collaborative TB/HIV activities: Guidelines for national programmes and other stakeholders; 2012. 23586124

[19] UCN/NCD/ISD W. Implementation Guidance to INTEGRATE Noncommunicable Disease Services into Other Programmatic Areas and Health Systems: Draft; 2021.

[20] WHO. Strengthening diagnostics capacity, Seventy-sixth World Health Assembly. In: WHA76.5, editor. 2023.

[21] Church K, Wringe A, Lewin S, Ploubidis GB, Fakudze P, Integra I, et al. Exploring the Feasibility of Service Integration in a Low-Income Setting: A Mixed Methods Investigation into Different Models of Reproductive Health and HIV Care in Swaziland. PloS One. 2015; 10(5). DOI: 10.1371/journal.pone.0126144 PMC4433110 25978632

[22] Jagosh J. Realist Synthesis for Public Health: Building an Ontologically Deep Understanding of How Programs Work, For Whom, and In Which Contexts. Annu Rev Public Health. 2019; 40: 361–72. DOI: 10.1146/annurev-publhealth-031816-044451 30633712

[23] Pawson R, Greenhalg, Harvey G, Walshe, K. Realist review – a new method of systematic review designed for complex policy interventions. Journal of Health Services Research & Policy. 2005; 10: 21–34. DOI: 10.1258/1355819054308530 16053581

[24] Rycroft-Malone J, McCormack B, Hutchinson A, DeCorby K, Bucknall T, Kent B, et al. Realist synthesis: illustrating the method for implementation research. Implementation Science. 2012; 7. DOI: 10.1186/1748-5908-7-33 22515663 PMC3514310

[25] Wong G, Greenhalgh T, Westhorp G, Buckingham J, Pawson, R. RAMESES publication standards: realist syntheses. BMC Medicine; 2013. DOI: 10.1186/1741-7015-11-21 PMC3558331 23360677

[26] Briggs CJ, Capdegelle P, Garner P. Strategies for integrating primary health services in middle- and low-income countries: effects on performance, costs and patient outcomes. Cochrane Database Syst Rev. 2001(4): CD003318. DOI: 10.1002/14651858.CD003318 11687187

[27] Adeyemi O, Lyons M, Njim T, Okebe J, Birungi J, Nana K, et al. Integration of non-communicable disease and HIV/AIDS management: a review of healthcare policies and plans in East Africa. BMJ Glob Health. 2021; 6(5). DOI: 10.1136/bmjgh-2020-004669 PMC8098934 33947706

[28] Youssef A, Chaudhary Z, Wiljer D, Mylopoulos M. Mapping Evidence of Patients’ Experiences in Integrated Care: A Scoping Review. General Hospital Psychiatry. 2019; 61. DOI: 10.1016/j.genhosppsych.2019.08.004 31479842

[29] Legido-Quigley H, Montgomery CM, Khan P, Atun R, Fakoya A, Getahun H, et al. Integrating tuberculosis and HIV services in low- and middle-income countries: a systematic review. Tropical Medicine and International Health. 2013; 18(2): 199–211. DOI: 10.1111/tmi.12029 23217030

[30] Gera T, Shah D, Garner P, Richardson M, Sachdev HS. Integrated management of childhood illness (IMCI) strategy for children under five. Cochrane Database Syst Rev. 2016(6): CD010123. DOI: 10.1002/14651858.CD010123.pub2 27378094 PMC4943011

[31] McCombe G, Lim J, Hout MCV, Lazarus JV, Bachmann M, Jaffar S, et al. Integrating Care for Diabetes and Hypertension with HIV Care in Sub-Saharan Africa: A Scoping Review. Int J Integr Care. 2022; 22(1): 6. DOI: 10.5334/ijic.5839 PMC8815447 35136387

[32] Duffy M, Ojikutu B, Andrian S, Sohng E, Minior T, Hirschhorn L. Non-communicable diseases and HIV care and treatment: models of integrated service delivery. Tropical Medicine and International Health. 2017; 22: 926–37. DOI: 10.1111/tmi.12901 28544500

[33] Kane J, Landess M, Carroll C, Nolen A, Sodhi S. A systematic review of primary care models for non-communicable disease interventions in Sub-Saharan Africa. BMC Family Practice. 2017; 18(46). DOI: 10.1186/s12875-017-0613-5 PMC5363051 28330453

[34] van Olmen J, Schellevis F, Van Damme W, Kegels G, Rasschaert F. Management ofChronic Diseases in Sub-Saharan Africa: Cross-Fertilisation between HIV/AIDS and Diabetes Care. Journal ofTropical Medicine; 2012. DOI: 10.1155/2012/349312 PMC3508584 23209477

[35] Hope R, Kendall T, Langer T, Barningausen T. Health Systems Integration of Sexual and Reproductive Health and HIV Services in Sub-Saharan Africa: A Scoping Study. J Acquir Immune Defic Syndr. 2014; 67. DOI: 10.1097/QAI.0000000000000381 25436826 PMC4251913

[36] Jongh T, I G-U, Allen EZ, NJ, Atun R. Barriers and enablers to integrating maternal and child health services to antenatal care in low and middle income countries. BJOG An International Journal of Obstetrics and Gynaecology; 2016. DOI: 10.1111/1471-0528.13898 PMC4768640 26861695

[37] Kikuchu K, Ayer R, Okawa S, Nishikitani M, Yokota F, Jimba M, et al. Interventions integrating non-communicable disease prevention and reproductive, maternal, newborn, and child health: A systematic review. BioScience Trends. 2018; 12(2): 116–25. DOI: 10.5582/bst.2018.01070 29760355

[38] WHO. Integrated Health Services – What and Why ?; 2008.

[39] Mwanahamuntu MH, Sahasrabuddhe VV, Pfaendler KS, Mudenda V, Hicks ML, Vermund SH, et al. Implementation of ‘see-and-treat’ cervical cancer prevention services linked to HIV care in Zambia. AIDS. 2009; 23(6): N1–5. DOI: 10.1097/QAD.0b013e3283236e11 19279439 PMC2747794

[40] Parham GP, Mwanahamuntu MH, Sahasrabuddhe VV, Westfall AO, King KE, Chibwesha C, et al. Implementation of cervical cancer prevention services for HIV-infected women in Zambia: measuring program effectiveness. HIV Ther. 2010; 4(6): 703–22. DOI: 10.2217/hiv.10.52 25419240 PMC4237284

[41] Church K, Warren CE, Birdthistle I, Ploubidis GB, Tomlin K, Zhou W, et al. Impact of Integrated Services on HIV Testing: A Nonrandomized Trial among Kenyan Family Planning Clients. Stud Fam Plann. 2017; 48(2): 201–18. DOI: 10.1111/sifp.12022 28470971 PMC5518195

[42] Gengiah S, Barker PM, Yende-Zuma N, Mbatha M, Naidoo S, Taylor M, et al. A cluster-randomized controlled trial to improve the quality of integrated HIV-tuberculosis services in primary healthcareclinics in South Africa. J Int AIDS Soc. 2021; 24(9): e25803. DOI: 10.1002/jia2.25803 34498370 PMC8426757

[43] Bygrave H, Golob L, Wilkinson L, Roberts T, Grimsrud A. Let’s talk chronic disease: can differentiated service delivery address the syndemics of HIV, hypertension and diabetes? Curr Opin HIV AIDS. 2020; 15(4): 256–60. DOI: 10.1097/COH.0000000000000629 32398467

[44] Ramogola-Masire D, de Klerk R, Monare B, Ratshaa B, Friedman HM, Zetola NM. Cervical cancer prevention in HIV-infected women using the “see and treat” approach in Botswana. J Acquir Immune Defic Syndr. 2012; 59(3): 308–13. DOI: 10.1097/QAI.0b013e3182426227 22134146 PMC3884088

[45] Maina W. Integrating noncommunicable disease prevention into maternal and child health programs: Can it be done and what will it take? International Journal of Gynecology and Obstetrics. 2011; 115. DOI: 10.1016/S0020-7292(11)60010-6 22099439

[46] Wroe EB, Kalanga N, Dunbar EL, Nazimera L, Price NF, Shah A, et al. Expanding access to non-communicable disease care in rural Malawi: outcomes from a retrospective cohort in an integrated NCD-HIV model. BMJ Open. 2020; 10(10): e036836. DOI: 10.1136/bmjopen-2020-036836 PMC7580053 33087368

[47] UNAIDS. Chronic care of HIV and noncommunicable diseases: How to leverage the HIV experience; 2011.

[48] Raben D, Hoekstra M, Combs L, Sullivan A, Lazarus JV, Lambert J, et al. A call to action toward integrated testing and earlier care for viral hepatitis, HIV, STIs and TB. HIV Medicine. 2020; 21: 403–8. DOI: 10.1111/hiv.12844

[49] Smith PJ, Davey DJ, Green H, Cornell M, Bekker LG. Reaching underserved South Africans with integrated chronic disease screening and mobile HIV counselling and testing: A retrospective, longitudinal study conducted in Cape Town. PLoS One. 2021; 16(5): e0249600. DOI: 10.1371/journal.pone.0249600 33945540 PMC8096085

[50] Doherty T, Chopra M, Tomlinson M, Oliphant N, Nsibande D, Mason J. Moving from vertical to integrated child health programmes: experiences from a multi-country assessment of the Child Health Days approach in Africa. Tropical Medicine and International Health. 2010; 15: 296–305. DOI: 10.1111/j.1365-3156.2009.02454.x 20070638

[51] Conference AotXIA. Track E Implementation Science, Health Systems and Economics. Journal of the International AIDS Society. 2012; 15(Suppl 3). DOI: 10.7448/IAS.15.5.18443

[52] Havlir DV, Balzer LB, Charlebois ED, Clark TD, Kwarisiima D, Ayieko J, et al. HIV Testing and Treatment with the Use of a Community Health Approach in Rural Africa. N Engl J Med. 2019; 381(3): 219–29. DOI: 10.1056/NEJMoa1809866 31314966 PMC6748325

[53] Young N, Achieng F, Desai M, Phillips-Howard P, Hill J, Aol G, et al. Integrated point-of-care testing (POCT) for HIV, syphilis, malaria and anaemia at antenatal facilities in western Kenya: a qualitative study exploring end-users’ perspectives of appropriateness, acceptability and feasibility. BMC Health Serv Res. 2019; 19(1): 74. DOI: 10.1186/s12913-018-3844-9 30691447 PMC6348645

[54] Bryce J, Victora CG, Habicht JP, Black RE, Scherpbier RW. Programmatic pathways to child survival: results of a multi-country evaluation of Integrated Management of Childhood Illness. Oxford University Press in association with The London School of Hygiene and Tropical Medicine; 2005. DOI: 10.1093/heapol/czi055 16306070

[55] Kjærgaard J, Anastasaki M, Østergaard MS, Isaeva E, Akylbekov A, Nguyen NQ, et al. Diagnosis and treatment of acute respiratory illness in children under five in primary care in low-, middle-, and high-income countries: A descriptive FRESH AIR study. PLoS ONE. 2019; 14(11). DOI: 10.1371/journal.pone.0221389 PMC6834279 31693667

[56] Shayo EH, Kivuyo S, Seeley J, Bukenya D, Karoli P, Mfinanga SG, et al. The acceptability of integrated healthcare services for HIV and non-communicable diseases: experiences from patients and healthcare workers in Tanzania. BMC Health Serv Res. 2022; 22(1): 655. DOI: 10.1186/s12913-022-08065-4 35578274 PMC9112557

[57] Mulupi S, Ayakaka I, Tolhurst R, Kozak N, Shayo E, Abdalla E, et al. Perspectives of healthcare workers, national and regional policy stakeholders on the management of chronic lung disease in five sub-saharan African countries: tale of a vicious cycle of neglect. Preprint; 2022. 10.1136/bmjopen-2021-052105 PMC9345041 35906045

[58] Sweeny S, Obure C, Terris-Prestholt F, Darsamo V, Michaels-Igbokwe C, Muketo E, et al. The impact of HIV/SRH service integration on workload: analysis from the Integra Initiative in two African settings. Human Resources for Health. 2014; 12. DOI: 10.1186/1478-4491-12-42 25103923 PMC4130428

[59] Bryce J, Victora CG, Habicht JP, Vaughan JP, Black RE. The multi-country evaluation of the integrated management of childhood illness strategy: lessons for the evaluation of public health interventions. Am J Public Health. 2004; 94(3): 406–15. DOI: 10.2105/AJPH.94.3.406 14998804 PMC1448266

[60] Wang M, Boeke CE, Rioja MR, Maparo T, Banda C, Chavula C, et al. Feasibility and impact of near-point-of-care integrated tuberculosis/HIV testing in Malawi and Zimbabwe. AIDS. 2021; 35(15): 2531–7. DOI: 10.1097/QAD.0000000000003031 34310372

[61] Ndlovu Z, Fajardo E, Mbofana E, Maparo T, Garone D, Metcalf C, et al. Multidisease testing for HIV and TB using the GeneXpert platform: A feasibility study in rural Zimbabwe. PLoS One. 2018; 13(3): e0193577. DOI: 10.1371/journal.pone.0193577 29499042 PMC5834185

[62] Organisation WH. Considerations for Adoption and Use of Multidisease testing devices in Integrated Laboratory networks; 2017.

[63] Manosuthi W, Wiboonchutikul S, Sungkanuparph S. Integrated therapy for HIV and tuberculosis. AIDS Res Ther. 2016; 13: 22. DOI: 10.1186/s12981-016-0106-y 27182275 PMC4866405

[64] Phetlhu D, Bimerew M, Marie-Modeste R, Naidoo M, Igumbor J. Nurses’ Knowledge ofTuberculosis, HIV, and Integrated HIV/TB Care Policies in Rural Western Cape, South Africa. Journal of the Association of Nurses in Aids Care. 2018; 29. DOI: 10.1016/j.jana.2018.05.008 29945760

[65] WHO. Monitoring the building blocks of health systems: A handbook of indicators and their measurement strategies. World Health Organisation; 2010.

[66] Tapela N, Tshisimogo G, Shatera BP, Letsatsi V, Gaborone M, Madidimalo T, et al. Integrating noncommunicable disease services into primary health care, Botswana. Bull World Health Organ. 2019; 97: 142–53. DOI: 10.2471/BLT.18.221424 30728620 PMC6357568

[67] Torpey K, Iwelunmor J, Ezechi O, Obiezu-Umeh C, Gbajabiamila T, Musa AZ, et al. Capabilities, opportunities and motivations for integrating evidence-based strategy for hypertension control into HIV clinics in Southwest Nigeria. Plos One. 2019; 14(6). DOI: 10.1371/journal.pone.0217703 PMC6553742 31170220

[68] Singer SJ, Kerrissey M, Friedberg M, Phillips R. A Comprehensive Theory of Integration. Medical Care Research and Review. 2018; 77(2): 196–207. DOI: 10.1177/1077558718767000 29606036

[69] Kabatereine NB, Malecela M, Lado M, Zaramba S, Amiel O, Kolaczinski JH. How to (or not to) integrate vertical programmes for the control of major neglected tropical diseases in sub-Saharan Africa. PLoS Negl Trop Dis. 2010; 4(6): e755. DOI: 10.1371/journal.pntd.0000755 20614017 PMC2894133

[70] de Jongh TE, Gurol-Urganci I, Allen E, Jiayue Zhu N, Atun R. Barriers and enablers to integrating maternal and child health services to antenatal care in low and middle income countries. BJOG. 2016; 123(4): 549–57. DOI: 10.1111/1471-0528.13898 26861695 PMC4768640

[71] Gwaza GP, Lamy M, Datta R, Dittrich S. Barriers to integrating diagnostic services for febrile illness to support surveillance and patient management in Asia-Pacific. Asia & the Pacific Policy Studies. 2022; 9(2): 196–212. DOI: 10.1002/app5.353

[72] Corneli A, Jarrett N, Sabue M, Duvall S, Bahati E, Behets F, et al. Patient and provider perspectives on implementation models of HIV counseling and testing for patients with TB. International Journal of Tuberculosis and Lung diseases. 2008; 12(3): 579–84. 18302828

[73] Zapata T, Forster N, Campuzano P, Kambapani R, Brahmbhatt H, Hidinua G, et al. How to Integrate HIV and Sexual and Reproductive Health Services in Namibia, the Epako Clinic Case Study. International Journal of Integrated Care. 2017; 17(4). DOI: 10.5334/ijic.2488 PMC5624130 28970759

[74] Gwaza G, Leqheka M, Dittrich S, Mots’oane T, Kao K. Missed opportunities for integrated testing: An Evaluation of the implementation of TB and HIV EID Testing on the GeneXpert platform in Lesotho. African Journal for Laboratory Science; 2023. DOI: 10.5334/ijic.ICIC23406 PMC10506622 37727530

[75] Chopra M, Binkin N, Mason E, Wolfheim C. Integrated management of childhood illness: what have we learned and how can it be improved? Global Child Health; 2012. DOI: 10.1136/archdischild-2011-301191 22278806

[76] Mitambo C, Khan S, Matanje-Mwagomba BL, Kachimanga C, Wroe E, Segula D, et al. Improving the screening and treatment of hypertension in people living with HIV: An evidence-based policy brief by Malawi’s Knowledge Translation Platform. Malawi Med J. 2017; 29(2): 224–8. DOI: 10.4314/mmj.v29i2.27 28955437 PMC5610300

[77] Youssef A, Wiljer D, Mylopoulos M, Maunder R, Sockalingam S. “Caring About Me”: a pilot framework to understand patient- centered care experience in integrated care – a qualitative study. BMJ Open. 2020; 10. DOI: 10.1136/bmjopen-2019-034970 PMC7389773 32718923

[78] CHAI. Integrated Testing for TB and HIV using GenExpert Devices Expands Access to Near-Point-of-Care Testing Lessons Learned from Zimbabwe; August 2019.

[79] Hyle P, Naidoo K, Su A, El-Sadr M, Freedberg A. HIV, Tuberculosis, and Non-Communicable Diseases: What is known about the costs, effects, and cost-effectiveness of integrated care? J Acquir Immune Defic Syndr. 2014; 67(1): S87–S95. DOI: 10.1097/QAI.0000000000000254 25117965 PMC4147396

[80] Sweeny S, Obure C, Terris-Presholt F, Darsamo V, Michaels-Igbokwe C, Muketo E, et al. The impact of HIV/SRH service integration on workload: analysis from the Integra Initiative in two African settings. Human Resources for Health. 2014; 12(42). DOI: 10.1186/1478-4491-12-42 PMC4130428 25103923

[81] Storeng K, Behague D. “Lives in the balance”: The politics of integration in the Partnership for Maternal, Newborn and Child Health. Health Policy and Planning. 2016; 31: 992–1000. DOI: 10.1093/heapol/czw023 27106911 PMC5013778

[82] Brodersen J, Schwartz L, Heneghan C, O’Sullivan J, Aronson J, Woloshin S. Overdiagnosis: what it is and what it isn’t. BMJ Evidence-Based Medicine. 2018; 23(1). DOI: 10.1136/ebmed-2017-110886 29367314

