# Supplementary material for: Integrated Diagnosis in Africa’s Low- and Middle-Income Countries: What Is It, What Works, and for Whom? A Realist Synthesis
Source: Int J Integr Care. 2024 Sep 12;24(3):20. doi: 10.5334/ijic.7788 (PMC11396343; doi:10.5334/ijic.7788)
Supplement: Supplementary file 1. — List of primary studies included in the review. [file ijic-24-3-7788-s1.pdf]

Supplementary file 1

List of primary studies included in the review.

Study title	Authors	Country	Study Design	Diseases integrated
1. Feasibility and impact of near-point-of-care integrated tuberculosis/HIV testing in Malawi and Zimbabwe	Wang et al., 2021	Malawi, Zimbabwe	Implementation study	HIV and TB
**2.** Multi-disease testing for HIV and TB using the GeneXpert platform: A feasibility study in rural Zimbabwe.	Ndlovu 2018	Zimbabwe	Implementation study	HIV and TB
3. The substantial burden of non-communicable diseases amongst adults: Screening results from an integrated testing services clinic for adults in Soweto, South Africa	Hopkins et, 2021	South Africa	Cross-sectional study	HIV and NCDs
4. Integrated point-of-care testing (POCT) for HIV, syphilis, malaria, and anaemia at antenatal facilities in western Kenya: a qualitative study exploring end-users 'perspectives of appropriateness, acceptability and feasibility	Young et al., 2019	Kenya	Qualitative study	HIV, syphilis, malaria, and anaemia
5. Perspectives of healthcare workers, national and regional policy stakeholders on the management of chronic lung disease in five sub-Saharan African countries: tale of a vicious cycle of neglect.	Mulupi et al, 2022	Kenya, Malawi, Sudan, Tanzania, and Uganda	Qualitative study	Chronic lung diseases
6. The association between a detectable HIV viral load and non-communicable diseases comorbidity in HIV positive adults on antiretroviral therapy in Western Cape, South Africa.	George et al, 2019	South Africa	Unclear	HIV and NCDs
7. Implementation of 'see-and-treat' cervical cancer prevention services linked to HIV care in Zambia.	Mwanahamuntu et al, 2009	Zambia	Implementation study	HIV and Cervical cancer
8. Implementation of cervical cancer prevention services for HIV-infected women in Zambia: measuring program effectiveness.	Parham et al, 2010	Zambia	Implementation study	HIV and Cervical cancer
9. Early experiences in integrating cervical cancer screening and treatment into HIV services in Zomba Central Hospital, Malawi	Pfaff et al, 2018	Malawi	Implementation study	HIV and Cervical cancer
10. Integrating HIV and hypertension management in low-resource settings: Lessons from Malawi.	Patel et al, 2018	Malawi	Implementation study	HIV and hypertension
11. Cervical cancer prevention in HIV-infected women using the "see and treat" approach in Botswana.	Ramogola-Masire, 2012	Botswana	Implementation study	HIV and Cervical cancer
12. The acceptability of integrated healthcare services for HIV and non-communicable diseases: experiences from patients and healthcare workers in Tanzania.	Shayo et al, 2022	Tanzania	Qualitative study	HIV and NCDs

13. Community Satisfaction with Primary Health Care Services. An evaluation undertaken in the Morogoro region of Tanzania	Gilson et al, 1994	Tanzania	Qualitative study	Primary care services
14. Patient and provider perspectives on implementation models of HIV counselling and testing for patients with TB	Corneli et al, 2008	Democratic Republic of Congo	Qualitative study	HIV and TB
15. Programmatic pathways to child survival: results of a multi-country evaluation of Integrated Management of Childhood Illness	Bryce et al, 2005	Bangladesh, Brazil, Peru, Tanzania, and Uganda.	Program evaluation	Integrated Management of childhood illnesses
16. Integrated primary health care in low- and middle-income countries: a double challenge.	Druetz, 2018	Burkina Faso	Program evaluation	Primary care and Malaria
17. Diagnosis and treatment of acute respiratory illness in children under five in primary care in low-, middle-, and high-income countries: A descriptive FRESH AIR study	KjærgaardI et al, 2019	Greece, Kyrgyzstan, Vietnam, and Uganda	Observational study	Acute respiratory diseases
18. Multimorbidity and care for hypertension, diabetes and HIV among older adults in rural South Africa	Chang et al, 2019	South Africa	Longitudinal study	Hypertension, diabetes and HIV
19. A cluster-randomized controlled trial to improve the quality of integrated HIV-tuberculosis services in primary healthcare clinics in South Africa.	Gengiah et al, 2021	South Africa	Cluster-randomized controlled trial	HIV and TB
20. Exploring the Feasibility of Service Integration in a Low-Income Setting: A Mixed Methods Investigation into Different Models of Reproductive Health and HIV Care in Swaziland.	Church et al, 2015	Swaziland	Comparative case study	HIV and reproductive health
21. Impact of Integrated Services on HIV Testing: A Non-randomized Trial among Kenyan Family Planning Clients	Church et al, 2017	Kenya	Non-randomized Trial	HIV and family planning
22. Expanding access to non-communicable disease care in rural Malawi: outcomes from a retrospective cohort in an integrated NCD-HIV model.	Wroe et al, 2020	Malawi	Retrospective cohort	HIV and NCDs
23. Reaching underserved South Africans with integrated chronic disease screening and mobile HIV counselling and testing: A retrospective, longitudinal study conducted in Cape Town.	Smith et al, 2021	South Africa	Retrospective, longitudinal study	Chronic diseases including HIV
24. The impact of HIV/SRH service integration on workload: analysis from the Integra Initiative in two African settings.	Sweeny et al, 2014	Kenya and Swaziland	Program Evaluation	HIV and SRH
25. How to Integrate HIV and Sexual and Reproductive Health Services in Namibia, the Epako Clinic Case Study	Zapata et al, 2017	Namibia	Observational study	HIV and SRH

List of Reviews included in this realist review

	Title	Author	Focus of integration
1	Strategies for integrating primary health services in low- and middle-income countries at the point of delivery	Dudley et al 2011	General Health services integration
2	Strategies for integrating primary health services in middle- and low-income countries: effects on performance, costs and patient outcomes	Briggs et al., 2001	General Health services integration
3	Integration of non-communicable disease and HIV/AIDS management: a review of healthcare policies and plans in East Africa	Adeyemi et al, 2021	NCDs and HIV/AIDS
4	Mapping Evidence of Patients' Experiences in Integrated Care: A Scoping Review	Youssef et al., 2019	General Health services integration
5	Integrating tuberculosis and HIV services in low- and middle-income countries: a systematic review.	Legido-Quigley et al, 2013	TB and HIV
6	Integrated management of childhood illness (IMCI) strategy for children under five.	Gera et al., 2016	Integrated Management of Childhood Illnesses (IMCI)
7	Integrating cardiovascular diseases, hypertension, and diabetes with HIV services: a systematic review	Haldane et al., 2017	Cardiovascular diseases, hypertension, diabetes and HIV
8	Integration of HIV/AIDS and non-communicable diseases in developing countries: rationale, policies and models.	Haregu et al., 2015	HIV/AIDS and NCDs
9	Integrating Care for Diabetes and Hypertension with HIV Care in Sub-Saharan Africa: A Scoping Review.	McCombe et al, 2022	Diabetes, Hypertension and HIV
10	Non-communicable diseases and HIV care and treatment: models of integrated service delivery.	Duffy et al., 2017	NCDs and HIV
11	A systematic review of primary care models for non-communicable disease interventions in Sub-Saharan Africa.	Kane et al., 2017	NCDs
12	Health Systems Integration of Sexual and Reproductive Health and HIV Services in Sub-Saharan Africa: A Scoping Study.	Hope et al., 2014	SRH and HIV
13	Management of Chronic Diseases in Sub-Saharan Africa: Cross-Fertilization between HIV/AIDS and Diabetes Care.	Ov et al., 2012	HIV/AIDS and Diabetes
14	Barriers and enablers to integrating maternal and child health services to antenatal care in low- and middle-income countries.	Jongh et al., 2016	Maternal and child health
15	Interventions integrating non-communicable disease prevention and reproductive, maternal, newborn, and child health: A systematic review	Kikuchu et al., 2018	NCDs, reproductive and maternal health
